# Mucinous cystadenocarcinoma of the ovary in a 14-year-old girl: a case report and literature review

**DOI:** 10.1186/s12905-023-02551-5

**Published:** 2023-07-21

**Authors:** Qiong Li, Cancan Zou, Yangyang Xu, Shiqing Liu, Tianjing Yan

**Affiliations:** 1 Department of Pathology, Chongqing Health Center for Women and Children, Women and Children’s Hospital of Chongqing Medical University, No. 120 Longshan Rd, Yubei District, Chongqing, 401147 China; 2 Department of Radiology, Chongqing Health Center for Women and Children, Women and Children’s Hospital of Chongqing Medical University, Chongqing, 401147 China; 3 Department of Ultrasound, Chongqing Health Center for Women and Children, Women and Children’s Hospital of Chongqing Medical University, Chongqing, 401147 China

**Keywords:** Ovarian epithelial tumor, Mucinous cystadenocarcinoma, Children, Case Report

## Abstract

**Background:**

Ovarian epithelial tumors are common in adults, and their peak incidence of onset is over 40 years of age. In children, most ovarian tumors are germ cell-derived, whereas epithelial tumors are rare and mostly benign.

**Case presentation:**

This report describes a case of a 14-year-old Chinese girl with ovarian mucinous cystadenocarcinoma. She was admitted with a small amount of bloody vaginal discharge during the past month. Magnetic resonance imaging of the abdomen and pelvis showed a large solid cystic mass lesion in the left ovary. Tumor marker levels were within normal limits ( CA-125: 22.3 U/mL, HE4: 28.5 pmol/L, HCG: < 1.20 mIU/ml, AFP: 3.3 ng/ml, CEA: 2.2 ng/ml, CA19-9: < 2.0 U/mL). Laparoscopic exploration revealed a large left ovarian tumor. The patient underwent left salpingo-oophorectomy, and showed no significant issues during follow-up, as well as no evidence of recurrence or metastasis.

**Conclusions:**

We report the first pediatric case of ovarian mucinous cystadenocarcinoma in China. Given the scarcity of reports addressing the clinical management of this condition, the present study provides a useful contribution to its further understanding in light of developing future treatment strategies.

## Background

The incidence of epithelial ovarian tumors in children is low, accounting for less than 20% among ovarian tumor types [1]. In addition, the vast majority of ovarian tumors in children are benign [2, 3], while malignant ovarian epithelial neoplasms are extremely rare. In this article, we report a case of a Chinese 14-year-old girl who developed ovarian mucinous cystadenocarcinoma after menarche. To our knowledge, this is the fifteenth reported case of cystadenocarcinoma (ninth case of mucinous cystadenocarcinoma) in children worldwide. The age range of the 14 previously reported cases identified in PubMed using the following search terms: ovary, epithelial carcinoma or tumor, mucinous carcinoma or tumor, adolescent, children or pediatric, was 4–14 years, with eight cases of mucinous cystadenocarcinoma and six cases of other cancer types (Table 1).

**Table 1:** Ovarian cystadenocarcinoma reported in the literature in < 15 years of age

Case No.	**Study**	Age(yr)	Pre/postmenarchal	Operative	Histology	**Adjuvant**	Outcom
				treatment		therapy	
1	Hong et al. (1980) [4]	4	Pre	Right salphingo-	Serous	Methotrexate,	No recurrence after 7 months, Long term follow-up not available
				oopherectomy		cyclophosphamide,	
						cis-diamminedicholoroplatinum	
2	Blom et al. (1982) [5]	4	Pre	Right salphingo-	Serous	Intra peritoneal instillation of	No recurrence after 4 months, Long term follow-up not available
				oopherectomy		32phosphorus	
3	Hernandez et al.(1982) [6]	10	Pre	Left salphingo- oopherectomy	Serous	Not given	No recurrence after 1.8 years,Longterm follow-up not available
4	Gribbon et al. (1992) [7]	NA	NA	Salphingo-	Serous	Pelvic radiotherapy	Disease free 17 years after diagnosis
				oopherectomy			
5	Gribbon et al. (1992) [7]	NA	NA	Salphingo- oopherectomy	Mucinous	Pelvic radiotherapy	Disease free 24 years after diagnosis
6	Skinner et al. (1992) [8]	13	NA	Salphingo- oopherectomy	Mucinous	Not given	Disease free 6 years after diagnosis
7	Shankar et al. (2001) [9]	7	Pre	Cytoreductive	Serous	Cyclophosphamide,	Died 12 years after diagnosis
				surgery		cis-diamminedicholoroplatinum	
8	Shankar et al. (2001) [9]	8	Pre	Left Salphingo-	Mucinous	Etoposide, carboplatin,bleomycin	In terminal care on last follow-up
				oopherectomy			
9	Shankar et al. (2001) [9]	5	Pre	Cytoreductive	Serous	cis-diamminedicholoroplatinum	In terminal care on last follow-up
				surgery			
10	Morowitz et al.(2003) [10]	NA	Pre	NA	Mucinous	Given	Died 1 years after diagnosis
11	Morowitz et al.(2003) [10]	NA	Post	Left Salphingo-	Mucinous	Not given	Disease free till last follow-up
				oopherectomy			
12	Morowitz et al.(2003) [10]	NA	Post	Salphingo-	Mucinous	Not given	Disease free till last follow-up
				oopherectomy			
13	Barkha et al. (2017) [1]	14	Post	Left Salphingo-	Mucinous	Cisplatin, paclitaxel	Disease free till last follow-up
				oopherectomy			
14	Agnieszka et al. (2022) [11]	14	Post	Left Salphingo-	Mucinous	Cisplatin, paclitaxel	Disease free till last follow-up
				oopherectomy			
15	Current study	14	Post	Left Salphingo-	Mucinous	Not given	Disease free till last follow-up
				oopherectomy			

## Case presentation

**Fig. 1:**
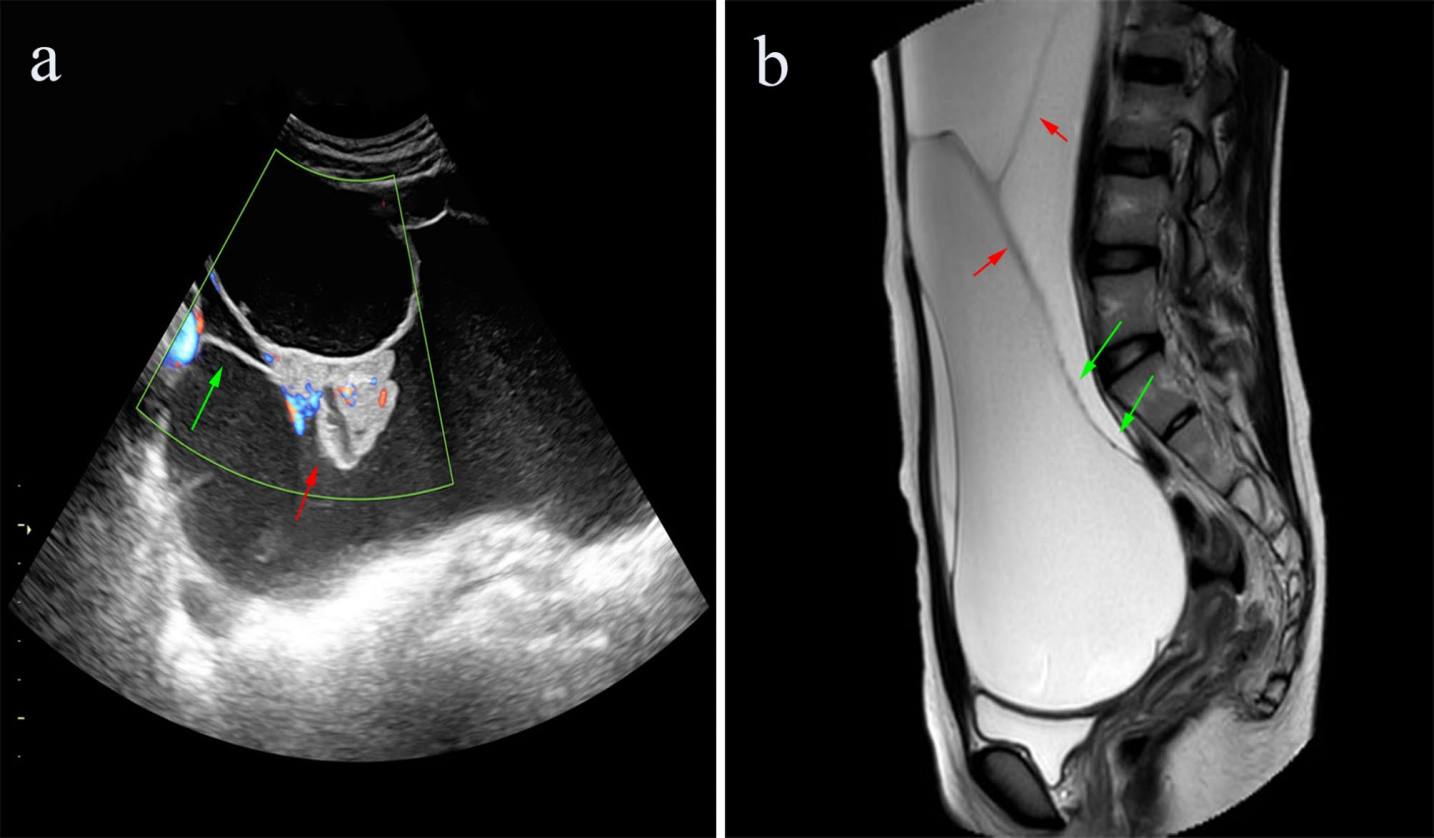
**(a)** Pelvic color Doppler ultrasound scan revealed a large mass, predominantly cystic, with poor internal echogenicity. It presented as fine, weakly echogenic spots, with numerous unevenly sized septations (green arrow), and several differently sized solid echogenic protrusions into the cystic cavity (red arrow), giving a cauliflower-like appearance. **(b)** MRI images revealed a large cystic-solid mass, predominantly cystic, with visible septations (red arrow) and wall nodules (green arrow) within

A 14-year-old girl was admitted to the gynecology outpatient department of Women and Children’s Hospital of Chongqing Medical University in April 2022, with complaints of a small amount of bloody vaginal discharge during the past month. Before this event she reported an average menstrual cycle of 25 days, with her menarche at the age of thirteen, and her periods averaging five days, with normal volume and no dysmenorrhea. In addition, the patient’s past medical history showed no history of infectious or genetic diseases, trauma, surgery, blood transfusion, drug or food allergies. Pelvic color doppler ultrasound scan revealed a mass with cystic and solid components in the left ovary extending upward to the inferior hepatic rim, downward to below the pubic symphysis, and forward to the anterior axillary line. Magnetic resonance imaging (MRI) scans of the abdomen and pelvis showed a large solid cystic mass lesion in the left ovary that measured 6.7 × 18.9 × 29.3 cm, and the contrasted scan showed an enhanced septum and solid component (Fig. 1). The liver and intrahepatic ducts, gallbladder, pancreas, spleen, both kidneys and ureters were unremarkable. The patient’s pre-operative serum levels showed that carbohydrate antigen (CA-125): 22.3 U/mL (normal: < 35 U/mL), human epididymal protein-4 (HE4): 28.5 pmol/L (normal: < 70 pmoI/L), human chorionic gonadotropin (HCG): < 1.20 mIU/ml (normal: < 5.0 mIU/ml), alpha-fetoprotein (AFP): 3.3 ng/ml (normal: 0–7 ng/ml), carcinoembryonic antigen (CEA): 2.2 ng/ml (normal: 0–5 ng/ml), carbohydrate antigen 19 − 9 (CA19-9): < 2.0 U/mL (normal: 0–43 U/mL) were within normal limits. The patient, with a height of 155 cm and weight of 45.5 kg (BMI: 18.9), presented no particular concerns regarding nutritional status, general vital signs, psychosocial state, or risk factors for stress-related injuries. The results of complete hemogram ( WBC: 7.5 × 10^9^/L, RBC: 4.2 × 10^12^/L, PLT: 216 × 10^9^/L, HGB: 127 g/L, et al. ) in addition to liver and kidney function tests ( TBIL:10.5 μm/L, DBIL: 3.4 μm/L, IBIL: 7.1 μm/L, ALB: 45 g/L, GLO: 20 g/L, A/G: 2.3, ALT: 13 U/L, AST: 20 U/L, UREA: 3.25 mmol/L, Cr: 56 umol/L, UA: 323 umol/L, et al. ) and coagulation profile ( APTT: 27.9 s, PT: 12 s, INR: 1.01, TT: 18 s, FIB: 2.7 g/L, FDP: <2 mg/L, D-D: 0.31 mg/L FEU ) were also normal.

After examination, given the patient’s pediatric status and the aim to minimize surgical invasiveness, the patient underwent laparoscopic adnexectomy (single-port surgery) which revealed a large left ovarian tumor measuring 29 × 25 × 15 cm with an intact capsule adhering to the omentum; however, the surrounding peritoneum and the right ovary appeared to be uneventful. A small amount of ascites was found in the abdominal cavity and the peritoneal lavage was sent for cytological examination. A left salpingo-oophorectomy was performed followed by frozen tissue sectioning. Subsequently, the patient underwent appendectomy and omentectomy, and the specimens were sent for routine histopathological examination. As no abnormalities were observed in the right ovary during the laparoscopic exploration, and in an effort to minimize surgical impact on the right ovarian function, we did not remove any part of the right ovary for pathological examination.

**Fig. 2:**
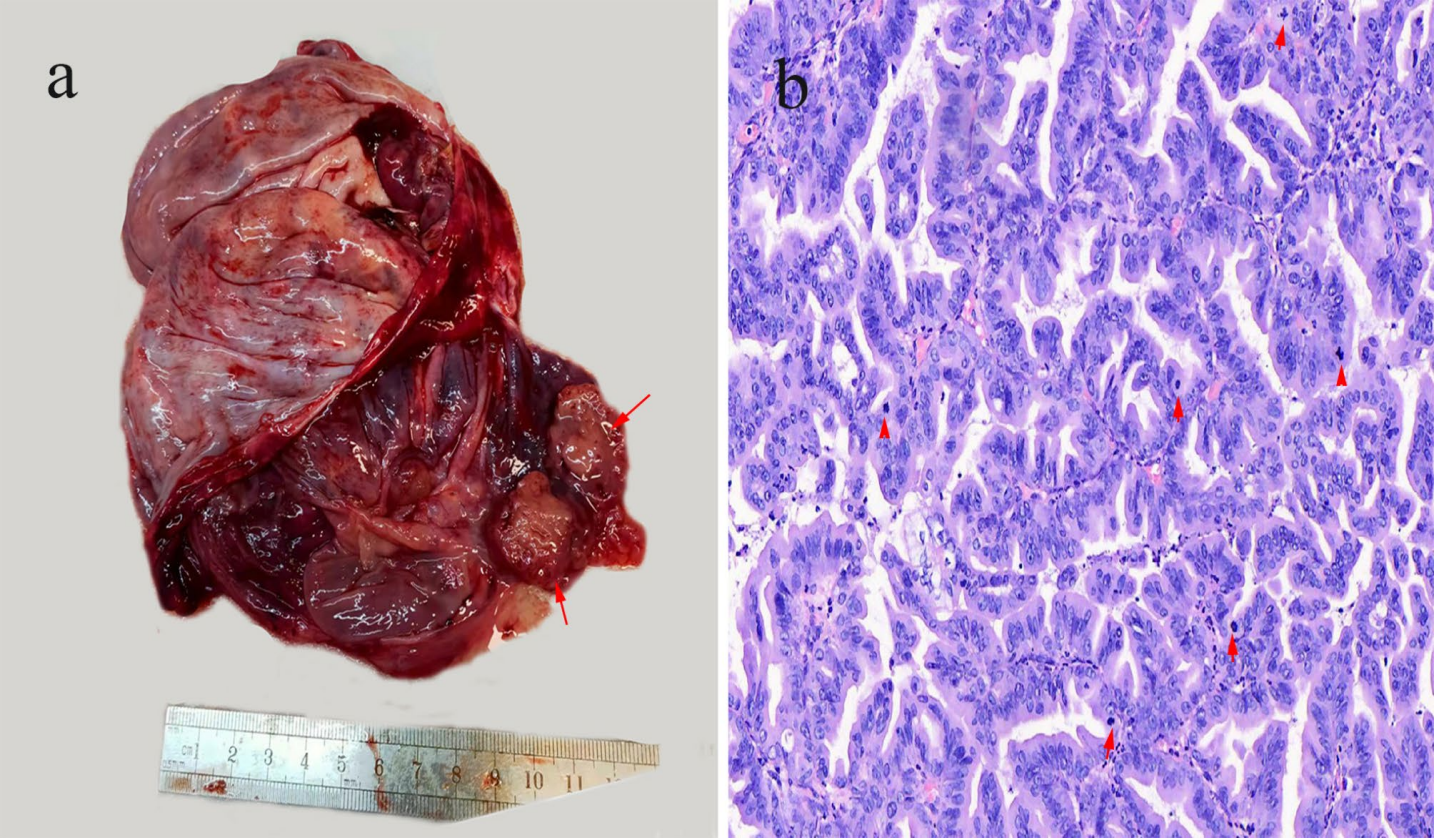
**(a)** Gross examination: The ovarian mass measured 15 × 9 × 4 cm, with a predominantly cystic appearance upon sectioning. The cysts were filled with turbid mucus, and their walls were largely smooth, with occasional surface irregularities and a pink nodular excrescence visible internally (red arrow). **(b)** Microscopic examination: The glands, exhibiting papillary and cribriform shapes, showed expansile growth with anastomosing architecture and minimal to absent stroma. The lining of most epithelial cells displayed moderate to severe atypia, with diminished or absent mucinous differentiation and conspicuous mitotic figures (red arrow) (H&E, x400)

Frozen sections revealed an enlarged dissected ovary measuring 15 × 9 × 4 cm, with a cystic cut section and presence of solid areas. Cysts were filled with turbid mucus, and their walls were mostly smooth with a few surface irregularities and a 3 × 2.5 × 1.5 cm pink nodular excrescence in the interior (Fig. 2a). No significant abnormalities were found in the fallopian tubes.

Microscopically, frozen sectioning followed by routine histological examination revealed a continuum of architectural and cytological atypia that included benign, borderline, and carcinomatous areas. Carcinomas with papillary and cribriform-shaped glands exhibited expansile growth, with anastomosing architecture and minimal or absent stroma; most of the epithelial cells were moderate to severely atypical in their lining, with reduced/absent mucinous differentiation and conspicuous mitoses. Based on these observations on the tissue pathological aspects, a diagnosis of mucinous cystadenocarcinoma with expansile invasion was finally made (Fig. 2b). The immunohistochemical staining results were as follows: ER-negative, PR-negative, CK7-diffuse positive, CK20-focal positive, CDX2-negative, SATB2-negative, GATA3-negative, Mammaglobin-negative:, GCDFP-15-negative, Pax8-negative, Vimentin-negative, Ki67: about 20% (+), WTI-negative, P53: wild-type expression.

For this patient, the levels of tumor markers, such as carbohydrate antigen (CA-125), human chorionic gonadotropin (HCG), alpha-fetoprotein (AFP), carcinoembryonic antigen (CEA), carbohydrate antigen 19 − 9 (CA19-9) and human epididymal protein-4 (HE4) were all within normal limits. The diagnosis of mucinous cystadenocarcinoma with expansile invasion was mainly based on imaging and pathology, especially pathology.

The patient was followed up after surgery every three months with gynecologic ultrasound and analysis of tumor markers (CEA, AFP, CA19-9, CA-125, HE4). Ultrasound results showed no pelvic mass and tumor markers were within normal range. Her current menstrual cycle is about 15 days, with an average period of 3–4 days, low volume, and no dysmenorrhea. The patient has so far shown no signs of recurrence and is still being followed up.

## Discussion

Here we reported a case of ovarian mucinous adenocarcinoma in a 14-year-old girl, which to our knowledge is the first case of ovarian epithelial tumor reported so far in Chinese and East Asian children. Of the eight cases of mucinous adenocarcinoma that have been previously reported in the literature worldwide, seven were from Europe or the United States, and one was from India. Some studies have suggested that ovarian cancer incidence may be related to race and ethnicity, with rates among white adolescents and young adults almost twice as high as those among black women of the same age group [12]. The question of whether racial and ethnic differences are also associated with the incidence of ovarian epithelial tumors in children has been so far unanswered, partly because of the few reported cases. Thus, continued attention and further research are needed in this regard.

In previous reports on mucinous carcinoma of the ovary in children under 15 years of age, the clinical and pathological features of the patients showed that most of the patients had abdominal pain and distention as their first symptoms; elevated serum tumor marker CA125 was reported in two cases, and mildly elevated HCG was reported in one case. None of these reports had follow-up of the patients’ postoperative menstrual status. In the present case, the patient did not have abdominal pain and distension, but presented with a small amount of bloody vaginal discharge. Through follow-up interviews, we know that her current menstrual cycle is about 15 days, with an average period of 3–4 days, low volume, and no dysmenorrhea.

Epithelial tumors of the ovary are very common in women, and most commonly occur during adulthood. Of note, the types of ovarian tumors occurring in children before the age of 15 differ from those diagnosed in adults, with a majority of sex cord-stromal and germ cell tumors, and less than 20% of epithelial tumors. In this line, Van et al. [13] reported ovarian masses in infancy, childhood and adolescence, with the incidence of epithelial and germ cell tumors being 15% and 70%, respectively in the under-15 age group, and 41% and 43% in the over-15 age group. Similar findings were obtained by Young et al. [12] and Li et al. [14].

Concerning ovarian cancer pathogenesis, the traditional view is that all ovarian cancer subtypes originate at the surface epithelium of the ovary. During ovulation, damage to the ovarian surface is caused by the follicle rupture and consequent oocyte release, and during damage repair, the epithelial cells on the ovarian surface become invaginated and form cortical cysts. Exposure of epithelial cells lining the cortical cyst to a new hormone-rich environment induces their proliferation, and eventually some epithelial cells that happen to harbor remaining DNA damage may become carcinogenic, thus leading to ovarian cancer [15]. This rationale explains those ovarian epithelial malignant tumors occurring after menarche but not the cases that occur before it. Consistently, the characteristics of ovarian epithelial tumors are different between children and adults, with serous tumors being the most common in adults while mucinous tumors are the most common in children. It has been suggested that some of the mucinous tumors in the age group of 10–14 may originate from monoblastic differentiation of the gastrointestinal mucinous epithelium in teratomas [16]. This is the age at which germ cell tumors are prone to occur, which may explain why a proportion of mucinous tumors occur before menarche.

The main mode of presentation of ovarian epithelial tumors was abdominal pain, bloating or menstrual disturbances with vague symptoms that were initially ignored. The girl reported in this case presented with a small amount of bloody vaginal discharge, which opportunely caught her attention. Notably, approximately 21% of patients with ovarian epithelial tumors are asymptomatic [14], which frequently leads to disease progression before the lesion is diagnosed.

Both analysis of tumor markers as well as radiological studies are important tools in the diagnosis of ovarian cancer [17]. CA 125 has been widely used as a marker for epithelial ovarian tumors, however, its positive predictive value is debatable. Although serum CA 125 levels (> 35 U/mL) are elevated in more than 80% of patients with ovarian epithelial carcinoma, they are also elevated in approximately 1% of non-neoplastic conditions such as endometriosis, cirrhosis, pancreatitis, pelvic inflammatory disease, and advanced abdominal -non-ovarian- malignancies [18]. Furthermore, serum CA 125 has been reported to have only 78.1% sensitivity and 76.8% specificity in detecting primary ovarian epithelial carcinoma [19]. In spite of this, it remains a useful tumor marker to be used in combination with imaging findings, which often show an adnexal mass in the presence of a tumor.

Frailty, characterized by increased vulnerability and reduced health response, plays an important role in predicting postoperative complications and survival outcomes in gynecological oncology. This assessment, which correlates with prolonged hospital stay and increased risk of organ failure, mortality, and rehospitalization, should be performed using standard scores such as the Clinical Frailty Scale (CFS-7) and Frailty Index (FI), [20]. This enables personalized therapeutic strategies for patients with gynecological malignancies, thereby improving oncological outcomes. Although we assessed her body mass index (BMI), nutritional status, general vital signs, psychosocial status, and risk factors for stress-related injury and concluded that she was likely to tolerate surgery with minimal risk of serious complications, we did not use specific frailty measures (e.g., CFS-7, FI) We recognize the importance of these assessments and acknowledge this as an area for future improvement.

Following surgery, the patient developed a low-grade fever, and complete blood count (CBC) indicated an elevated white blood cell count and neutrophil percentage, warranting antibiotic treatment. Postoperative pain was managed with pain management therapy, resulting in only mild discomfort, as indicated by a Visual Analogue Scale (VAS) score of less than 3. No other postoperative complications were observed.

The prognosis of ovarian cancers presenting at a young age is variable and depends on the tumor stage upon presentation as well as histological type. In the eight reported cases of ovarian mucinous carcinoma in children to date, five were at stage I. Among these, one case was found to have cancerous thrombi in the vessels and died from recurrent metastasis two years later, while the rest achieved a survival time of over five years. The other three cases were at a higher stage: one died a year after diagnosis, and the other two had either metastasis at the time of diagnosis or cancer cells were found in the ascites, suggesting a less favorable prognosis.

Treatment of ovarian epithelial carcinoma in children relies on the experience from treating adult patients, with an emphasis on the preservation of reproductive functions. However, individualized regimens have been developed based on the establishment of comprehensive surgical staging. In patients with low-grade stage IA (serous, endometrioid or mucinous expansile subtype), fertility-sparing surgery (FSS) appears to be a safe option [21–23]. FSS can also be considered for stage IC1 tumors, as recurrences, which are often isolated on the remaining ovary, can typically be managed with subsequent surgery. However, it’s important to note that recurrence rates tend to be higher in stage IC2, IC3, and grade 3 diseases, mainly in extra-ovarian sites. This suggests that these recurrences may not be directly associated with the fertility-sparing approach. Therefore, comprehensive counseling is crucial when considering FSS in these situations [24].

Various adjuvant chemotherapy regimens have been used in epithelial carcinoma of the ovary to improve survival. A comprehensive cohort study [25] found that chemotherapy not only reduced mortality for high-risk patients but also for those with stage IA/IB, grade 2 ovarian cancer. This aligns with previous studies showing no advantage of chemotherapy for women with stage IA and IB, grade 1 tumors. In particular, for histological subtypes like mucinous subtype, the expansile or grade I type, which is linked to a better prognosis, is not recommended for adjuvant chemotherapy, while the infiltrative form has a high relapse risk [26–29].

## Conclusions

In summary, ovarian epithelial tumors are very rare in children, and their pathogenesis, especially before menarche, remains unclear, which poses a challenge to the treatment and management of the disease. In this context, the present case report adds valuable information concerning the clinical, serological and imaging characteristics of mucinous cystadenocarcinoma, a rare type of epithelial ovarian cancer, in a young patient. Given the scarcity of reports addressing the clinical management of this condition, the present study provides a useful contribution to its further understanding in light of developing future treatment strategies.

## Data Availability

The datasets analyzed during the current study are available from the corresponding author on reasonable request.

## References

[1] Gupta B, Orcid Id, Arora P, Khurana N, Tempe A (2017). Mucinous cystadenocarcinoma of ovary with metastasis in 14-year-old girl. Obstet Gynecol Sci.

[2] Biçer S, Erkul Z, Demiryilmaz I, Peker N (2014). A 9-kg ovarian mucinous cystadenoma in a 14-year-old premenarchal girl. Am J Case Rep.

[3] Karaman A, Azili MN, Boduroğlu EC, Karaman I, Erdoğan D, Cavuşoğlu YH (2008). A huge ovarian mucinous cystadenoma in a 14-year-old premenarchal girl: review on. J Pediatr Adolesc Gynecol.

[4] Hong SJ, Lurain JR, Tsukada Y, Piver MS, Humbert JR, Freeman AI (1980). Cystadenocarcinoma of the ovary in a 4-year-old: benign transformation during. Cancer.

[5] Blom GP, Torkildsen EM (1982). Ovarian cystadenocarcinoma in a 4-year-old girl: report of a case and review of. Gynecol Oncol.

[6] Hernandez E, Rosenshein NB, Parmley TH (1982). Mucinous cystadenocarcinoma in a premenarchal girl. South Med J.

[7] Gribbon M, Ein SH, Mancer K (1992). Pediatric malignant ovarian tumors: a 43-year review. J Pediatr Surg.

[8] Skinner MA, Schlatter MG, Heifetz SA, Grosfeld JL (1993). Ovarian neoplasms in children. Arch Surg.

[9] Shankar KR, Wakhlu A, Kokai GK, McDowell H, Jones MO (2001). Ovarian adenocarcinoma in premenarchal girls. J Pediatr Surg.

[10] Morowitz M, Huff D, von Allmen D (2003). Epithelial ovarian tumors in children: a retrospective analysis. J Pediatr Surg.

[11] Drosdzol-Cop A, Mizia-Malarz A, Wilk K, Koszutski T, Wilk K, Stojko R (2022). Ovarian adenocarcinoma in 14-year-old girl. Ginekol Pol.

[12] Young JL, Cheng Wu X, Roffers SD, Howe HL, Correa C, Weinstein R. Ovarian cancer in children and young adults in the United States, 1992–1997. Cancer. 2003;97 Suppl:2694 – 700. 10.1002/cncr.11351 12733134

[13] Van Winter JT, Simmons PS, Podratz KC (1994). Surgically treated adnexal masses in infancy, childhood, and adolescence. Am J Obstet Gynecol.

[14] Li M, Pan LY, Huang HF, Lang JH (2004). Epithelial ovarian tumors in adolescence: a study of clinical features and. Zhonghua fu chan ke za zhi.

[15] Wong AS, Leung PC (2007). Role of endocrine and growth factors on the ovarian surface epithelium. J Obstet Gynaecol Res.

[16] Morris HB, La Vecchia C, Draper GJ (1984). Malignant epithelial tumors of the ovary in childhood: a clinicopathological. Gynecol Oncol.

[17] Deprest J, Moerman P, Corneillie P, Ide P (1992). Ovarian borderline mucinous tumor in a premenarchal girl: review on ovarian. Gynecol Oncol.

[18] Zanaboni F, Vergadoro F, Presti M, Gallotti P, Lombardi F, Bolis G (1987). Tumor antigen CA 125 as a marker of ovarian epithelial carcinoma. Gynecol Oncol.

[19] Leake J, Woolas RP, Daniel J, Oram DH, Brown CL (1994). Immunocytochemical and serological expression of CA 125: a clinicopathological. Histopathology.

[20] Ottavia, Dʼoria et al. Tullio Golia D’Augè,. The role of preoperative frailty assessment in patients affected by gynecological cancer: a narrative review. Ital J Gynaecol Obstet 2022, 34, N.2.

[21] Bentivegna E, Gouy S, Maulard A (2016). Fertility-sparing surgery in epithelial ovarian cancer: a systematic review of oncological issues. Ann Oncol.

[22] Satoh T, Hatae M, Watanabe Y (2010). Outcomes of fertility-sparing surgery for stage I epithelial ovarian cancer: a proposal for patient selection. J Clin Oncol.

[23] Fruscio R, Corso S, Ceppi L (2013). Conservative management of early-stage epithelial ovarian cancer: results of a large retrospective series. Ann Oncol.

[24] Bentivegna E, Fruscio R, Roussin S (2015). Long-term follow-up of patients with an isolated ovarian recurrence after conservative treatment of epithelial ovarian cancer: review of the results of an international multicenter study comprising 545 patients. Fertil Steril.

[25] Chatterjee S, Chen L, Tergas AI (2016). Utilization and outcomes of chemotherapy in women with intermediate-risk, early-stage ovarian cancer. Obstet Gynecol.

[26] Lee KR, Scully RE (2000). Mucinous tumors of the ovary: a clinicopathologic study of 196 borderline tumors (of intestinal type) and carcinomas, including an evaluation of 11 cases with ‘pseudomyxoma peritonei. Am J Surg Pathol.

[27] Gouy S, Saidani M, Maulard A (2017). Staging surgery in early-stage ovarian mucinous tumors according to expansile and infiltrative types. Gynecol Oncol Rep.

[28] Muyldermans K, Moerman P, Amant F (2013). Primary invasive mucinous ovarian carcinoma of the intestinal type: importance of the expansile versus infiltrative type in predicting recurrence and lymph node metastases. Eur J Cancer.

[29] Prat J, De Nictolis M (2002). Serous borderline tumors of the ovary: a long-term follow-up study of 137 cases, including 18 with a micropapillary pattern and 20 with microinvasion. Am J Surg Pathol.

